# BMI gain and dietary characteristics are risk factors of MASLD in non-obese individuals

**DOI:** 10.1038/s41598-025-86424-x

**Published:** 2025-01-21

**Authors:** Hirokazu Taniguchi, Miho Ueda, Yukiko Kobayashi, Takatomo Shima

**Affiliations:** 1 Division of Applied Life Sciences, Graduate School of Life and Environmental Sciences, Kyoto Prefectural University, Kyoto, 606-8522 Japan; 2 Center for Health Promotion, Japanese Red Cross Kyoto Daiichi Hospital, Kyoto, Japan

**Keywords:** MASLD, NAFLD, SLD, Non-obesity, Vegetables, Soy products, Non-alcoholic fatty liver disease, Dyslipidaemias

## Abstract

This longitudinal observational study aimed to evaluate whether cardiometabolic factors and dietary characteristics are determinants of metabolic dysfunction-associated steatotic liver disease (MASLD) in non-obese individuals (body mass index [BMI] < 25 kg/m²). The study was conducted at the Japanese Red Cross Society Kyoto Daiichi Hospital. Clinical data were longitudinally recorded at annual health checks. The diagnosis of MASLD was based on the results of abdominal ultrasonography and cardiometabolic criteria. Lifestyle behaviors and dietary characteristics were assessed using a self-administered questionnaire. A total of 4,100 non-obese middle-aged and older participants (1,636 men and 2,464 women) were followed up for an average of 6.44 ± 4.16 years. During the follow-up period, there were 410 new cases of MASLD in men (25.1%) and 484 in women (19.6%). The incidence rate was higher for men (39.7 per 1,000 person-years) than for women (30.1 per 1,000 person-years). Multivariable-adjusted logistic regression analyses using the rate of change per year with standardized values found that BMI gain was strongly associated with the onset of MASLD for both men (OR: 1.90, 95% CI: 1.64–2.19) and women (OR: 1.95, 95% CI: 1.72–2.21). Increased waist circumference and triglycerides were also associated with MASLD onset for both men and women. Lowering of high-density lipoprotein cholesterol was identified as a risk factor for MASLD in both men and women. Regarding dietary characteristics, the onset of MASLD was significantly and negatively associated with “often eating vegetables” for men (OR: 0.73, 95% CI: 0.57–0.93) and “often eating soy products” for women (OR: 0.71, 95% CI: 0.58–0.88), even after adjusting for BMI change and other covariates. These findings suggest that maintaining body weight and favorable dietary characteristics are key factors in the prevention of MASLD in non-obese individuals.

**Supplementary Information:**

The online version contains supplementary material available at 10.1038/s41598-025-86424-x.

## Introduction

Steatotic liver disease (SLD) is a condition characterized by excessive hepatic fat accumulation^1,2^. The presence of fatty liver without significant alcohol consumption is diagnosed as non-alcoholic fatty liver disease (NAFLD)^3^. NAFLD is the leading cause of chronic liver diseases^4,5^, cardiovascular diseases^6^, and mortality^7^. Recently, an international consensus panel has proposed that the term NAFLD be replaced by metabolic dysfunction-associated steatotic liver disease (MASLD), which includes cardiometabolic criteria such as obesity, dysglycemia, hypertension, and dyslipidemia^1,2^. Recent studies have reported that mortality rates were similarly increased by MASLD^8,9^. Therefore, further studies are required to elucidate the independent factors of MASLD among the criteria and the difference between NAFLD and MASLD.

The major risk of SLD is obesity^10^; however, NAFLD is widely prevalent in non-obese individuals (defined as body mass index [BMI] < 25 kg/m^2^)^11–14^. Non-obese NAFLD patients had worse outcomes of comorbidities and mortality than obese NAFLD patients^15,16^. Thus, the assessment of risk factors for non-obese SLD has important implications for the prevention of various diseases and reduction of mortality. Previous longitudinal studies reported that body weight gain was an independent risk factor for NAFLD among non-obese adults^17–19^. Nevertheless, whether the longitudinal changes in other cardiometabolic criteria of MASLD are associated with the risk of non-obese SLD remains to be elucidated.

Various guidelines recommend a healthy lifestyle, including physical activity and dietary factors, to prevent NAFLD^20–23^, and previous cross-sectional studies have reported that unhealthy dietary characteristics are associated with the prevalence of non-obese NAFLD^24–26^. Because unhealthy lifestyle factors are predictors of weight gain^27,28^, the evaluation of dietary characteristics associated with MASLD needs to account for longitudinal changes in body weight. However, there is a lack of longitudinal evidence on the association between dietary characteristics and MASLD in non-obese individuals.

The purpose of this study was to determine cardiometabolic and dietary factors of MASLD onset among non-obese individuals in a longitudinal observational study, thereby identifying independent factors of non-obese MASLD. Secondary analyses were conducted to determine if these associations were similar among non-obese individuals with NAFLD.

## Methods

### Study design and ethical approval

We performed a longitudinal observational study at Japanese Red Cross Society Kyoto Daiichi Hospital in Kyoto city, Japan. The baseline data of SLD were described in our previous cross-sectional study^26^. The clinical data were longitudinally recorded in annual health checks (Ningen dock), which are voluntary comprehensive health checkups for early disease detection in Japan^29^. All procedures involving human participants were approved by the Ethical Committees of Japanese Red Cross Society Kyoto Daiichi Hospital (approval number: 874) and Kyoto Prefectural University (approval number: 182). This study was conducted according to the guidelines laid down in the Declaration of Helsinki. Opt-out informed consent was obtained from all participants.

### The diagnosis of SLD and MASLD criteria

The diagnosis of SLD was based on the results of abdominal ultrasonography with hepatorenal contrast and liver brightness performed by trained technicians. The final diagnosis was determined by a gastroenterologist or preventive medicine specialist in accordance with the established diagnostic guidelines of the Japan Society of Ultrasonics in Medicine^30^ and the Japan Society of Hepatology^21,22^. Liver brightness, vascular blurring, hepatorenal echo contrast, and deep attenuation were evaluated to make a final diagnosis of NAFLD^31,32^.

The criteria of MASLD were liver steatosis with at least one of five cardiometabolic risk factors^1,2^: (1) waist circumference (WC) > 94 cm in men and > 80 cm in women; (2) fasting glucose (FG) ≥ 100 mg/dL, hemoglobin A1c (HbA1c) ≥ 5.7%, type 2 diabetes, or treatment for type 2 diabetes; (3) systolic blood pressure (SBP) ≥ 130 mmHg, diastolic blood pressure (DBP) ≥ 85 mmHg, or specific antihypertensive drug treatment; (4) triglycerides (TG) ≥ 150 mg/dL or lipid-lowering treatment; and (5) high-density lipoprotein cholesterol (HDL-C) ≤ 40 mg/dL in men and ≤ 50 mg/dL in women or lipid-lowering treatment. Alcohol-related/associated SLD was defined as weekly consumption of alcohol in excess of 210 g for men and 140 g for women.

### Study population and exclusion criteria

**Fig. 1:**
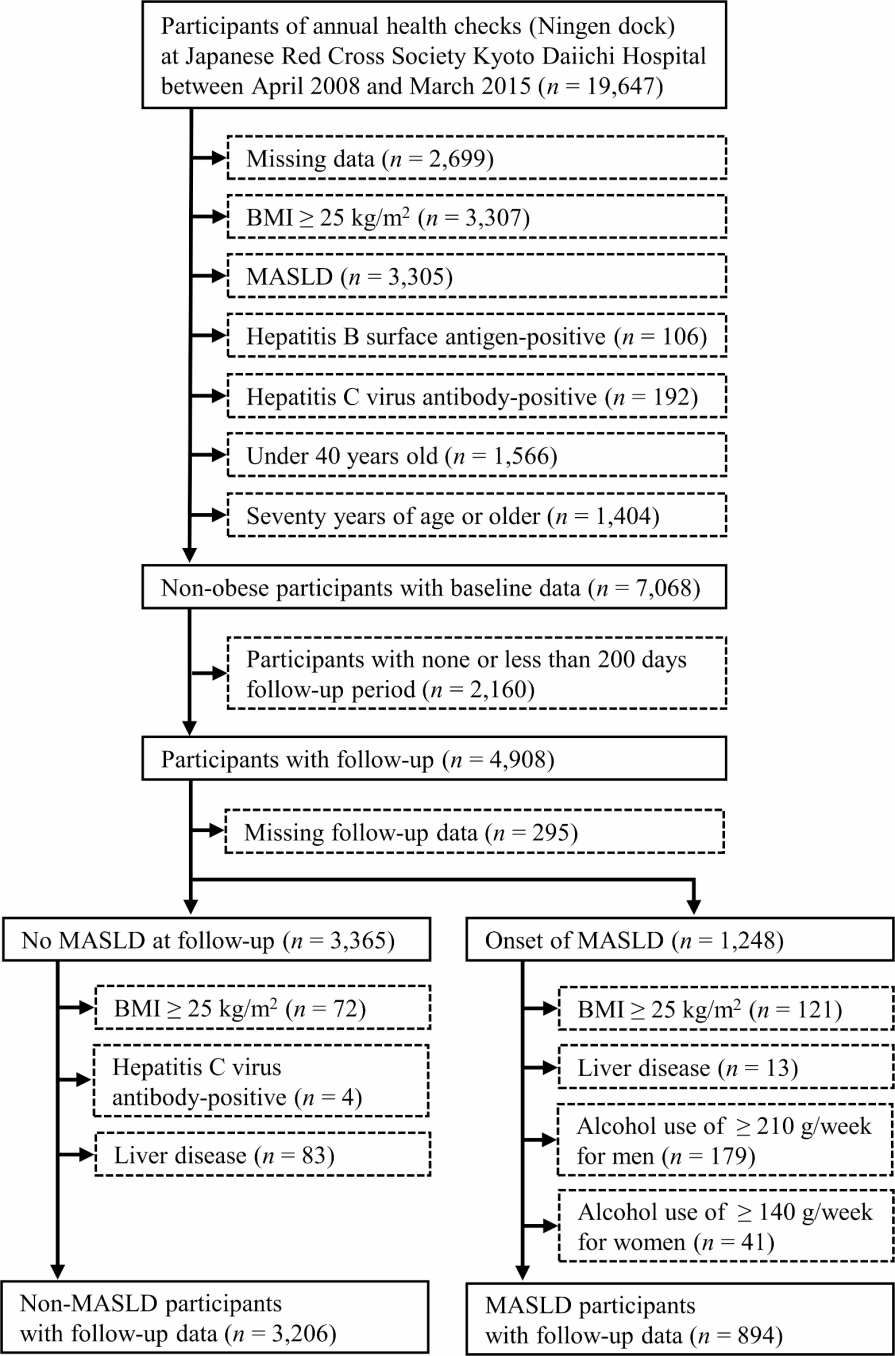
Flow diagram of the participants in the longitudinal study. BMI, body mass index; MASLD, metabolic dysfunction-associated steatotic liver disease.

The flow diagram of this study is shown in Fig. 1. The participants consisted of 19,647 adult individuals (aged 20–93 years old), and the baseline data were obtained at the first visit to the annual health check between April 2008 and March 2015. Missing data resulted in 2,699 being excluded at the baseline.

For the non-obese participants, other exclusion criteria at the baseline were as follows: BMI ≥ 25 kg/m^2^ (*n* = 3,307), MASLD (*n* = 3,305), B surface antigen-positive hepatitis (*n* = 106), C virus antibody-positive hepatitis (*n* = 192). Multiple lifestyle changes were predicted to be potential confounding factors in younger (such as marital events) and elderly (such as bereavement and/or cognitive decline) individuals. Thus, the study excluded individuals aged less than 40 years (*n* = 1,566) and those aged 70 years or above (*n* = 1,404). After the baseline collection, clinical data were obtained until March 2022, and 2,160 participants were excluded because of none or less than 200-days follow-up period. Follow-up was terminated if the participant had either new-onset MASLD or finished follow-up until March 2022. Participants with missing data at follow-up were excluded (*n* = 295). For participants with non-MASLD at follow-up (*n* = 3,365), exclusion criteria were as follows: BMI ≥ 25 kg/m^2^ (*n* = 72), C virus antibody-positive hepatitis (*n* = 4), and liver disease (*n*= 83). For liver disease, individuals with a history of liver disease and/or undergoing treatment were excluded using a self-administered questionnaire^33^. MASLD was diagnosed in 1,248 participants during the follow-up period. The data of non-obese participants with MASLD were obtained after exclusion of participants with BMI ≥ 25 kg/m^2^ (*n* = 121), liver disease (*n* = 13), and excessive alcohol use of ≥ 210 g/week for men and ≥ 140 g/week for women (*n*= 179 and 41, respectively)^1,2^. Finally, we analyzed 3,206 non-obese participants with non-MASLD and 894 non-obese participants with MASLD.

New-onset NAFLD was similarly followed up until March 2022 (Figure S1). Participants with SLD were excluded at the baseline (*n* = 3,659). The secondary analyses included data of 3,051 non-obese participants with non-NAFLD and 887 non-obese participants with NAFLD.

### Lifestyle and dietary questionnaires

Lifestyle behaviors were assessed using a self-administered questionnaire based on the Health Department of the Ministry of Health, Labour and Welfare in Japan^33^. The participants replied to questionnaires regarding their medical treatment including hypertension, hyperlipidemia, liver disease, and diabetes mellitus. Alcohol use was evaluated by asking the participants about the amount and type of alcoholic beverages consumed per week and used to estimate the mean ethanol intake per week. Habitual alcohol drinking was defined as consumption of more than 20 g/day ethanol and drinking alcohol more than three times a week, based on the National Health and Nutrition Survey in Japan^34^ and the general questionnaire^33,35^. Smoking habits were classified as never, former, and current smokers. Habitual physical activity was defined as physical activity for more than 1 h per day.

We included additional questions about dietary characteristics that are related to SLD and/or obesity^26,36,37^. Participants were asked about their eating habits, such as whether they often consumed seven food items (often eat vegetables, often eat fruits, often eat soy products, often eat sesame/nuts, often eat sweet buns/bread with fillings, often eat sweets, and often consume soft drinks), four types of food (often eat noodles/rice bowls, often eat stir-/deep-fried food, often eat simmered/teriyaki food, and often eat out/eat ready-made food), and three dietary behaviors (fast eating, often eat an evening meal, and consume ≥ 30 different food items per day). Applicability criteria for “often” and “consume ≥ 30 different food items per day” were almost every day.

### Measurements

Anthropometric and blood parameters were longitudinally measured from baseline to follow-up. Anthropometric measurements were performed as described previously^38^. BMI was calculated as body weight (kg) divided by the square of height (m). WC was measured twice in the standing position at the umbilicus. Venous blood samples were collected after at least 12 h of fasting, and chemical analysis was performed using standard techniques. Aspartate aminotransferase (AST), alanine aminotransferase (ALT), and gamma-glutamyl transpeptidase (γ-GTP) were measured as markers of liver injury. Lipid and glucose parameters were measured by TG, total cholesterol (TC), low-density lipoprotein cholesterol (LDL-C), HDL-C, FG, and HbA1c National Glycohemoglobin Standardization Program (NGSP) values, which were calculated according to the standard equation of the Japan Diabetes Society (JDS): HbA1c (NGSP) % = 1.02 × HbA1c (JDS) % + 0.25%^39^.

#### Statistical analyses

Analysis was performed for men and women separately because there are known sex differences in the prevalence of SLD^40^and MASLD-related dietary characteristics^26^. SPSS version 29.0 (SPSS Inc.) was used for statistical analysis. Continuous variables were presented as means ± standard deviations, and categorical variables were expressed as numbers (%). Significance was set at p-value < 0.05.

The person-years of follow-up were estimated for each sex. The incidence rate was presented as the number of MASLD cases per 1,000 person-years. Changes in anthropometric and blood measurements were calculated by subtracting baseline data from follow-up data and presented as delta values (⊿). Differences between the non-MASLD and MASLD groups were assessed using the Mann-Whitney U test (continuous variables) and Chi-square test (categorical variables).

Logistic regression model was used to estimate the odds ratio (OR) and 95% confidence interval (95% CI) of MASLD/NAFLD onset. The follow-up period was terminated at the onset of MASLD/NAFLD and was therefore shorter for participants with MASLD/NAFLD than those with non-MASLD/NAFLD. A logistic model was thus first constructed using the rate of change per year with z-score normalization as covariates. The rate of change was calculated by dividing the difference between the baseline and follow-up values by the baseline values. Subsequently, the rate of change was expressed as a percentage and divided by the follow-up years. The anthropometric and blood parameters of the MASLD criteria were standardized using z-score normalization, which was based on the mean rate of change and the standard deviation by sex. These associations were adjusted for baseline age, BMI, smoking habits, drinking habits, physical activity habits, and under medical treatment. Second, the baseline dietary characteristics were incorporated as covariates, and “not applicable” was used as a reference. Based on the association between body weight gain and the onset of NAFLD in non-obese adults^17–19^, these associations were adjusted for ⊿BMI, rather than baseline BMI, along with other baseline covariates (age, smoking habits, drinking habits, physical activity habits, and under medical treatment).

## Results

### New-MASLD onset in non-obese participants during the follow-up period

The average follow-up period for all participants was 6.44 ± 4.16 years, and the periods were 6.32 ± 4.24 years for men and 6.52 ± 4.10 years for women. This study included a total of 26,407 person-years of follow-up. The person-years were 10,337 years for men and 16,070 years for women. During the follow-up period, 894 (21.8%) new cases of MASLD were identified; therefore, the incidence rate was 33.9 per 1,000 person-years. New cases of MASLD were 410 (25.1%) in men and 484 (19.6%) in women. The incidence rate was higher for men (39.7 per 1,000 person-years) than for women (30.1 per 1,000 person-years).

### Anthropometric and blood parameters from baseline to follow-up in non-obese participants with non-MASLD and MASLD

Table 1 shows anthropometric and blood parameters at baseline, follow-up, and ⊿ values. For both men and women, ⊿age and ⊿SBP were significantly higher in the participants with non-MASLD than in those with new-onset MASLD. Both male and female participants with new-onset MASLD showed a significant increase in BMI and WC compared to non-MASLD participants. The increases in TG, ALT, and γ-GTP were significantly higher in participants with new-onset MASLD than non-MASLD. ⊿HDL-C was significantly higher in non-MASLD participants than new-onset MASLD participants in both men and women. Compared to non-MASLD women, ⊿LDL-C and ⊿HbA1c were significantly higher in women with onset of MASLD.

**Table 1:** Baseline and follow-up characteristics and comparison of changes in the clinical data between non-obese individuals with non-MASLD and MASLD.

Non-obese men (BMI < 25 kg/m^2^)	Non-MASLD (*n* = 1,226)			MASLD onset (*n* = 410)			*p*
	Baseline	Follow-up	⊿	Baseline	Follow-up	⊿	
Age, year	57.1 ± 8.4	64.2 ± 9.4	7.1 ± 4.3	55.2 ± 8.3	59.3 ± 8.8	4.1 ± 3.1	** < 0.001**
Height, cm	168.9 ± 6.0	168.3 ± 6.1	−0.7 ± 0.9	169.1 ± 5.8	168.8 ± 5.9	−0.3 ± 0.6	** < 0.001**
Weight, kg	60.4 ± 6.6	59.4 ± 7.0	−1.0 ± 3.3	62.6 ± 6.5	63.5 ± 6.5	1.0 ± 2.6	** < 0.001**
BMI, kg/m^2^	21.2 ± 1.9	21.0 ± 2.0	−0.2 ± 1.1	21.9 ± 1.7	22.3 ± 1.7	0.4 ± 0.9	** < 0.001**
WC, cm	79.8 ± 6.3	79.4 ± 6.4	−0.4 ± 4.5	81.8 ± 5.3	83.3 ± 5.5	1.5 ± 3.7	** < 0.001**
SBP, mmHg	124.3 ± 16.0	129.1 ± 17.8	4.8 ± 16.4	123.9 ± 15.4	125.5 ± 15.5	1.6 ± 14.8	** < 0.001**
DBP, mmHg	77.5 ± 10.1	77.1 ± 11.1	−0.4 ± 10.3	77.9 ± 10.0	77.0 ± 10.0	−0.9 ± 9.3	0.372
TG, mg/dL	89.7 ± 42.1	88.7 ± 43.7	−1.0 ± 41.8	107.3 ± 64.0	119.6 ± 70.9	12.3 ± 71.1	** < 0.001**
TC, mg/dL	203.1 ± 30.2	199.7 ± 30.8	−3.4 ± 26.9	207.7 ± 33.1	205.8 ± 32.1	−2.0 ± 29.0	0.249
LDL-C, mg/dL	116.0 ± 25.9	114.4 ± 26.9	−1.6 ± 24.3	123.3 ± 28.6	124.0 ± 29.3	0.8 ± 26.1	0.055
HDL-C, mg/dL	60.5 ± 14.5	66.7 ± 17.1	6.2 ± 11.0	55.4 ± 12.4	56.0 ± 13.5	0.6 ± 8.1	** < 0.001**
AST, IU/L	21.7 ± 8.1	22.3 ± 7.6	0.6 ± 8.9	21.0 ± 6.1	21.6 ± 5.9	0.5 ± 6.3	0.916
ALT, IU/L	18.9 ± 8.9	18.6 ± 8.1	−0.3 ± 9.4	20.4 ± 8.5	21.7 ± 10.3	1.4 ± 9.0	**0.002**
γ-GTP, IU/L	36.1 ± 36.5	34.2 ± 35.8	−1.9 ± 30.3	38.2 ± 36.0	41.3 ± 42.0	3.1 ± 24.4	** < 0.001**
FG, mg/dL	100.9 ± 16.2	103.6 ± 17.0	2.6 ± 11.8	101.6 ± 19.0	103.5 ± 15.2	1.8 ± 13.1	0.399
HbA1c, %	5.7 ± 0.6	5.8 ± 0.6	0.0 ± 0.5	5.8 ± 0.8	5.8 ± 0.6	0.0 ± 0.7	0.154

Non-obese women (BMI < 25 kg/m^2^)	Non-MASLD (*n* = 1,980)			MASLD onset (*n* = 484)			*p*
	Baseline	Follow-up	⊿	Baseline	Follow-up	⊿	
Age, year	56.0 ± 8.4	63.0 ± 9.2	7.0 ± 4.1	54.7 ± 8.1	59.2 ± 8.3	4.5 ± 3.2	** < 0.001**
Height, cm	156.5 ± 5.5	155.7 ± 5.7	−0.8 ± 1.1	156.9 ± 5.5	156.6 ± 5.5	−0.3 ± 0.6	** < 0.001**
Weight, kg	49.4 ± 5.6	48.8 ± 6.1	−0.6 ± 2.9	52.1 ± 5.2	53.4 ± 5.6	1.2 ± 2.5	** < 0.001**
BMI, kg/m^2^	20.2 ± 2.0	20.1 ± 2.1	0.0 ± 1.2	21.2 ± 1.7	21.7 ± 1.8	0.6 ± 1.0	** < 0.001**
WC, cm	76.2 ± 7.0	75.8 ± 7.1	−0.4 ± 5.3	79.2 ± 6.3	81.1 ± 6.3	1.9 ± 4.6	** < 0.001**
SBP, mmHg	118.7 ± 16.3	124.7 ± 18.5	6.0 ± 15.8	121.0 ± 16.7	123.2 ± 17.0	2.2 ± 14.8	** < 0.001**
DBP, mmHg	72.3 ± 10.6	71.7 ± 11.2	−0.6 ± 9.6	73.7 ± 10.7	72.4 ± 10.8	−1.3 ± 9.3	0.292
TG, mg/dL	78.1 ± 35.7	79.5 ± 37.7	1.4 ± 34.2	84.9 ± 36.8	96.3 ± 44.3	11.4 ± 37.1	** < 0.001**
TC, mg/dL	217.9 ± 32.9	218.4 ± 32.1	0.5 ± 32.5	214.9 ± 32.5	215.9 ± 32.8	1.0 ± 33.5	0.517
LDL-C, mg/dL	121.4 ± 28.1	123.8 ± 28.8	2.4 ± 28.2	122.9 ± 27.8	128.6 ± 29.5	5.7 ± 30.1	**0.012**
HDL-C, mg/dL	70.0 ± 14.4	77.8 ± 17.9	7.8 ± 12.5	65.1 ± 13.7	66.3 ± 14.4	1.2 ± 9.0	** < 0.001**
AST, IU/L	21.0 ± 13.1	21.9 ± 6.7	0.9 ± 13.3	19.9 ± 13.9	20.5 ± 5.2	0.6 ± 13.4	0.731
ALT, IU/L	16.1 ± 8.3	16.5 ± 7.9	0.4 ± 9.5	16.4 ± 16.6	17.5 ± 8.0	1.1 ± 15.6	** < 0.001**
γ-GTP, IU/L	20.9 ± 16.9	22.2 ± 20.3	1.3 ± 16.5	20.1 ± 17.5	22.4 ± 19.3	2.3 ± 14.2	** < 0.001**
FG, mg/dL	94.3 ± 11.0	98.0 ± 12.4	3.7 ± 9.7	95.8 ± 11.0	99.4 ± 13.1	3.6 ± 8.0	0.270
HbA1c, %	5.7 ± 0.4	5.7 ± 0.5	0.1 ± 0.5	5.7 ± 0.4	5.8 ± 0.5	0.1 ± 0.4	**0.002**

ALT, alanine aminotransferase; AST, aspartate aminotransferase; BMI, body mass index; DBP, diastolic blood pressure; FG, fasting glucose; γ-GTP, γ-glutamyl transpeptidase; HbA1c, hemoglobin A1c; HDL-C, high-density lipoprotein cholesterol; LDL-C, low-density lipoprotein cholesterol; MASLD, metabolic dysfunction-associated steatotic liver disease; SBP, systolic blood pressure; TC, total cholesterol; TG, triglycerides; WC, waist circumference.Data are the mean ± standard deviations. Mann–Whitney U test was calculated between non-MASLD and MASLD participants according to sex. Boldface indicates significance (*p* < 0.05).

### Baseline characteristics in non-obese participants with non-MASLD and MASLD

Dietary, lifestyle, and medical characteristics at baseline are shown in Table 2. For both men and women, non-MASLD participants were more likely to “often eat vegetables” and “soy products” compared to those with MASLD onset. Male participants with new-onset MASLD had a higher preference for “often consume soft drinks” and were more likely to “often eat an evening meal”. In the case of women with new-onset MASLD, the answer to “often eat sesame/nuts” was lower, while “often eat stir-fry/fried foods” was higher compared to non-MASLD women. Both male and female participants with new-onset MASLD had lower drinking habits than non-MASLD participants. Men without MASLD had a significantly higher prevalence of hypertension and being under medical treatment than men with new-onset MASLD.

### Association between MASLD criteria and new-onset MASLD in non-obese participants

For both non-obese men and women, univariate logistic regression analyses using the rate of change per year with standardized values found that BMI gain was most associated with new-onset MASLD (Table 3). The significant association remained strong both after adjusting for baseline age/BMI and multivariable covariates. The onset of MASLD was positively associated with WC and TG, whereas it was negatively associated with HDL-C in both sexes. For non-obese women, new-onset MASLD was positively associated with the rate of change in FG. Table S1 shows the rate of change per year in the MASLD criteria for non-obese men and women.

**Table 2:** Comparison of baseline data including dietary characteristics, lifestyle habits, and medical treatment between non-obese individuals with non-MASLD and MASLD.

	Non-obese men (BMI < 25 kg/m^2^)					Non-obese women (BMI < 25 kg/m^2^)				
	Non-MASLD (*n* = 1,226)		MASLD onset (*n* = 410)			Non-MASLD (*n* = 1,980)		MASLD onset (*n* = 484)		
	*n*	(%)	*n*	(%)	*p*	*n*	(%)	*n*	(%)	*p*
Food preferences										
Vegetables	**757**	**(61.7)**	215	(52.4)	** < 0.001**	**1452**	**(73.3)**	330	(68.2)	**0.023**
Fruits	392	(32.0)	118	(28.8)	0.227	878	(44.3)	193	(39.9)	0.076
Soy products	**515**	**(42.0)**	147	(35.9)	**0.028**	**959**	**(48.4)**	188	(38.8)	** < 0.001**
Sesame/nuts	165	(13.5)	64	(15.6)	0.277	**458**	**(23.1)**	87	(18.0)	**0.014**
Sweet buns/bread with fillings	250	(20.4)	82	(20.0)	0.865	421	(21.3)	106	(21.9)	0.759
Sweets	228	(18.6)	90	(22.0)	0.137	866	(43.7)	215	(44.4)	0.786
Soft drinks	128	(10.4)	**65**	**(15.9)**	**0.003**	145	(7.3)	36	(7.4)	0.931
Food styles										
Noodles/rice bowls	350	(28.5)	131	(32.0)	0.190	317	(16.0)	75	(15.5)	0.782
Stir-/deep-fried food	342	(27.9)	122	(29.8)	0.469	384	(19.4)	**116**	**(24.0)**	**0.025**
Simmered/teriyaki food	368	(30.0)	119	(29.0)	0.704	920	(46.5)	207	(42.8)	0.143
Eating out/ready-made food	263	(21.5)	90	(22.0)	0.831	291	(14.7)	87	(18.0)	0.073
Dietary behaviors										
Fast eating	647	(52.8)	227	(55.4)	0.362	858	(43.3)	233	(48.1)	0.056
Evening meal	314	(25.6)	**130**	**(31.7)**	**0.016**	684	(34.5)	172	(35.5)	0.681
Consume ≥ 30 foods per day	132	(10.8)	42	(10.2)	0.766	409	(20.7)	81	(16.7)	0.053
Smoking habits										
None	449	(36.6)	153	(37.3)	0.143	1740	(87.9)	417	(86.2)	0.222
Past	559	(45.6)	167	(40.7)		148	(7.5)	46	(9.5)	
Current	218	(17.8)	90	(22.0)		92	(4.6)	21	(4.3)	
Drinking habits	**568**	**(46.3)**	137	(33.4)	** < 0.001**	**200**	**(10.1)**	33	(6.8)	**0.016**
Physical activity habits	406	(33.1)	120	(29.3)	0.720	713	(36.0)	154	(31.8)	0.068
Hypertension	**185**	**(15.1)**	42	(10.2)	**0.021**	169	(8.5)	55	(11.4)	0.117
Dyslipidemia	78	(6.4)	25	(6.1)	0.987	211	(10.7)	66	(13.6)	0.117
Type 2 Diabetes	48	(3.9)	14	(3.4)	0.651	16	(0.8)	8	(1.7)	0.134
Any medical treatment	**297**	**(24.2)**	76	(18.5)	**0.036**	343	(17.3)	106	(21.9)	0.051

BMI, body mass index; MASLD, metabolic dysfunction-associated steatotic liver disease.

Chi-square test was calculated between non-MASLD and MASLD participants according to sex.

Data were presented as *n* (%). Boldface indicates significance (*p* < 0.05) and higher applicable percentage.

### Association between baseline dietary characteristics and new-onset MASLD in non-obese participants

Table 4 shows the results of the logistic regression analyses, which examined the association between baseline dietary characteristics and the onset of MASLD in non-obese men. The univariate analyses revealed that “often eat vegetables” and “soy products” were significantly and negatively associated with new-onset MASLD, whereas “often consume soft drinks” and “often eat an evening meal” were significantly and positively associated with new-onset MASLD. After adjusting for baseline age and ⊿BMI, the association of MASLD onset with vegetable intake and evening meal remained significant in the non-obese men, whereas the statistical significance disappeared for soy products and soft drinks. The multivariable-adjusted model that included ⊿BMI and baseline characteristics (smoking, physical activity, drinking, and medical treatment) found that “often eat vegetables” for men was a significant negative factor of new-onset MASLD.

**Table 3:** Association between rate of change per year in MASLD criteria and MASLD onset in non-obese Japanese men and women.

	Univariate			Baseline age and BMI adjusted			Multivariable adjusted*		
	OR	(95% CI)	*p*	OR	(95% CI)	*p*	OR	(95% CI)	*p*
Non-obese men (BMI < 25 kg/m^2^)									
Rate of change in BMI z-score (per year)	1.78	(1.55–2.03)	** < 0.001**	1.92	(1.66–2.22)	** < 0.001**	1.90	(1.64–2.19)	** < 0.001**
Rate of change in WC z-score (per year)	1.57	(1.39–1.78)	** < 0.001**	1.64	(1.44–1.86)	** < 0.001**	1.62	(1.42–1.85)	** < 0.001**
Rate of change in SBP z-score (per year)	1.04	(0.93–1.16)	0.545	1.07	(0.95–1.19)	0.272	1.06	(0.95–1.19)	0.319
Rate of change in DBP z-score (per year)	1.00	(0.90–1.12)	0.948	1.01	(0.90–1.13)	0.838	1.01	(0.91–1.13)	0.813
Rate of change in TG z-score (per year)	1.36	(1.21–1.52)	** < 0.001**	1.36	(1.21–1.52)	** < 0.001**	1.38	(1.24–1.55)	** < 0.001**
Rate of change in HDL-C z-score (per year)	0.78	(0.69–0.89)	** < 0.001**	0.77	(0.68–0.87)	** < 0.001**	0.76	(0.67–0.86)	** < 0.001**
Rate of change in FG z-score (per year)	1.08	(0.96–1.21)	0.183	1.06	(0.95–1.20)	0.299	1.07	(0.95–1.20)	0.274
Rate of change in HbA1c z-score (per year)	1.04	(0.94–1.15)	0.416	1.04	(0.94–1.16)	0.408	1.06	(0.95–1.17)	0.311
Non-obese women (BMI < 25 kg/m^2^)									
Rate of change in BMI z-score (per year)	1.88	(1.67–2.11)	** < 0.001**	1.95	(1.72–2.20)	** < 0.001**	1.95	(1.72–2.21)	** < 0.001**
Rate of change in WC z-score (per year)	1.53	(1.37–1.71)	** < 0.001**	1.51	(1.36–1.69)	** < 0.001**	1.52	(1.36–1.70)	** < 0.001**
Rate of change in SBP z-score (per year)	0.97	(0.88–1.07)	0.494	0.99	(0.90–1.09)	0.816	0.99	(0.90–1.09)	0.845
Rate of change in DBP z-score (per year)	1.00	(0.90–1.10)	0.932	1.01	(0.91–1.11)	0.892	1.00	(0.91–1.11)	0.976
Rate of change in TG z-score (per year)	1.24	(1.13–1.36)	** < 0.001**	1.22	(1.12–1.34)	** < 0.001**	1.22	(1.11–1.34)	** < 0.001**
Rate of change in HDL-C z-score (per year)	0.79	(0.71–0.87)	** < 0.001**	0.81	(0.73–0.89)	** < 0.001**	0.80	(0.72–0.89)	** < 0.001**
Rate of change in FG z-score (per year)	1.16	(1.05–1.28)	**0.004**	1.15	(1.04–1.27)	**0.006**	1.14	(1.03–1.26)	**0.010**
Rate of change in HbA1c z-score (per year)	1.04	(0.95–1.13)	0.397	1.03	(0.94–1.13)	0.549	1.03	(0.94–1.13)	0.507

*Adjusted for baseline age, BMI, smoking habit, physical activity habit, drinking habit, and medical treatment.

BMI, body mass index; CI, confidence interval; DBP, diastolic blood pressure; FG, fasting glucose; HbA1c, hemoglobin A1c; HDL-C, high-density lipoprotein cholesterol; MASLD, metabolic dysfunction-associated steatotic liver disease; OR, odds ratio; SBP, systolic blood pressure; TG, triglycerides; WC, waist circumference.

The results of non-adjusted and covariate-adjusted logistic regression are shown. Boldface indicates significance (*p* < 0.05).

In univariate logistic analyses of women (Table 5), there was a significant negative association between new-onset MASLD and “often eat vegetables”, “soy products”, and “sesame/nuts”, whereas there was a significant positive association between “often eat stir-/deep-fried food” and MASLD onset. The onset of MASLD was negatively associated with “often eat soy products” after adjusting for baseline age and ⊿BMI. After the adjustment, statistical significance was no longer observed for vegetables, sesame/nuts, and stir-/deep-fried food intake in the non-obese women. The multivariable-adjusted analyses found that “often eat soy products” for women was a significant negative factor of new-onset MASLD.

**Table 4:** Association between baseline dietary characteristics and MASLD onset in non-obese Japanese men.

	Non-obese men (BMI < 25 kg/m^2^)								
	Univariate			Baseline age and ⊿BMI adjusted			Multivariable adjusted*		
	OR	(95% CI)	*p*	OR	(95% CI)	*p*	OR	(95% CI)	*p*
Food preferences									
Vegetables	0.68	(0.55–0.86)	** < 0.001**	0.73	(0.57–0.92)	**0.008**	0.73	(0.57–0.93)	**0.009**
Fruits	0.86	(0.67–1.10)	0.227	0.98	(0.76–1.27)	0.870	0.94	(0.73–1.23)	0.669
Soy products	0.77	(0.61–0.97)	**0.028**	0.87	(0.68–1.10)	0.241	0.87	(0.68–1.12)	0.281
Sesame/nuts	1.19	(0.87–1.63)	0.278	1.27	(0.91–1.75)	0.156	1.28	(0.92–1.78)	0.142
Sweet buns/bread with fillings	0.98	(0.74–1.29)	0.865	0.93	(0.70–1.24)	0.602	0.89	(0.66–1.19)	0.416
Sweets	1.23	(0.94–1.62)	0.138	1.34	(1.00–1.78)	**0.047**	1.29	(0.96–1.72)	0.086
Soft drinks	1.62	(1.17–2.23)	**0.003**	1.35	(0.96–1.88)	0.084	1.22	(0.87–1.73)	0.250
Food styles									
Noodles/rice bowls	1.18	(0.92–1.50)	0.191	1.09	(0.85–1.39)	0.519	1.09	(0.85–1.41)	0.499
Stir-/deep-fried food	1.10	(0.86–1.40)	0.469	1.10	(0.85–1.41)	0.483	1.14	(0.88–1.48)	0.317
Simmered/teriyaki food	0.95	(0.75–1.22)	0.704	1.06	(0.81–1.37)	0.686	1.08	(0.83–1.41)	0.552
Eating out/ready-made food	1.03	(0.79–1.35)	0.832	0.96	(0.72–1.27)	0.761	0.96	(0.73–1.28)	0.803
Dietary behaviors									
Fast eating	1.11	(0.89–1.39)	0.362	1.12	(0.88–1.41)	0.358	1.10	(0.87–1.39)	0.410
Evening meal	1.35	(1.06–1.72)	**0.016**	1.30	(1.01–1.67)	**0.042**	1.21	(0.94–1.56)	0.145
Consume ≥ 30 foods per day	0.95	(0.66–1.37)	0.766	1.05	(0.72–1.53)	0.810	1.09	(0.75–1.61)	0.647

*Adjusted for ⊿BMI, baseline age, smoking habit, physical activity habit, drinking habit, and medical treatment.

BMI, body mass index; CI, confidence interval; MASLD, metabolic dysfunction-associated steatotic liver disease; OR, odds ratio.

The results of non-adjusted and covariate-adjusted logistic regression are shown. Boldface indicates significance (*p* < 0.05).

**Table 5:** Association between baseline dietary characteristics and MASLD onset in non-obese Japanese women.

	Non-obese women (BMI < 25 kg/m^2^)								
	Univariate			Baseline age and ⊿BMI adjusted			Multivariable adjusted*		
	OR	(95% CI)	*p*	OR	(95% CI)	*p*	OR	(95% CI)	*p*
Food preferences									
Vegetables	0.78	(0.63–0.97)	**0.023**	0.86	(0.69–1.08)	0.194	0.86	(0.68–1.08)	0.188
Fruits	0.83	(0.68–1.02)	0.076	0.92	(0.74–1.14)	0.421	0.91	(0.73–1.13)	0.383
Soy products	0.68	(0.55–0.83)	** < 0.001**	0.72	(0.58–0.88)	**0.002**	0.71	(0.58–0.88)	**0.002**
Sesame/nuts	0.73	(0.57–0.94)	**0.015**	0.78	(0.60–1.02)	0.064	0.79	(0.61–1.03)	0.078
Sweet buns/bread with fillings	1.04	(0.82–1.32)	0.759	1.00	(0.78–1.29)	0.979	0.98	(0.77–1.27)	0.902
Sweets	1.03	(0.84–1.26)	0.786	1.04	(0.84–1.27)	0.727	1.01	(0.82–1.25)	0.920
Soft drinks	1.02	(0.70–1.49)	0.931	0.83	(0.56–1.23)	0.360	0.81	(0.54–1.20)	0.291
Food styles									
Noodles/rice bowls	0.96	(0.73–1.27)	0.782	0.98	(0.74–1.30)	0.879	1.01	(0.76–1.35)	0.923
Stir-/deep-fried food	1.31	(1.03–1.66)	**0.025**	1.23	(0.96–1.57)	0.104	1.23	(0.96–1.58)	0.100
Simmered/teriyaki food	0.86	(0.71–1.05)	0.144	0.93	(0.75–1.15)	0.509	0.93	(0.75–1.16)	0.515
Eating out/ready-made food	1.27	(0.98–1.66)	0.073	1.11	(0.85–1.46)	0.446	1.12	(0.85–1.48)	0.407
Dietary behaviors									
Fast eating	1.21	(1.00–1.48)	0.057	1.22	(0.99–1.49)	0.063	1.20	(0.97–1.47)	0.088
Evening meal	1.05	(0.85–1.29)	0.681	1.00	(0.80–1.23)	0.966	0.98	(0.79–1.21)	0.835
Consume ≥ 30 foods per day	0.77	(0.59–1.00)	0.053	0.88	(0.67–1.15)	0.352	0.89	(0.68–1.17)	0.416

*Adjusted for ⊿BMI, baseline age, smoking habit, physical activity habit, drinking habit, and medical treatment.

BMI, body mass index; CI, confidence interval; MASLD, metabolic dysfunction-associated steatotic liver disease; OR, odds ratio.

The results of non-adjusted and covariate-adjusted logistic regression are shown. Boldface indicates significance (*p* < 0.05).

### Association between new-onset NAFLD and the related variables in non-obese participants

The results of secondary analyses about NAFLD onset are shown in Tables S2 and S3. The factors associated with the onset of NAFLD were largely consistent with those associated with the onset of MASLD in non-obese individuals. In the anthropometric and blood parameters (Table S2), multivariable-adjusted analyses using the rate of change per year with standardized values found that BMI gain was strongly and positively associated with new-onset NAFLD in both men (OR 1.87, 95% CI 1.62–2.16) and women (OR 1.84, 95% CI 1.63–2.07). The rate of change in WC, TG, and HDL-C were significantly associated with new-onset MASLD in both men and women. Regarding dietary characteristics (Table S3), the adjusted models showed that new-onset NAFLD was significantly and negatively associated with “often eat vegetables” among men (OR 0.64, 95% CI 0.50–0.82) and “soy products” among women (OR 0.69, 95% CI 0.56–0.86).

## Discussion

The present study performed a longitudinal observational study to assess the determinant factors of MASLD in non-obese middle-aged and older adults. For MASLD criteria, the onset of non-obese MASLD was strongly associated with BMI gain, and it was also associated with WC, TG, and HDL-C changes during follow-up. Moreover, the risk of MASLD was decreased by favorable dietary characteristics (vegetable intake for men, and soy intake for women). These results suggest that body weight management and favorable dietary characteristics are important for preventing MASLD onset in non-obese individuals.

The criteria of MASLD include SLD and cardiometabolic risk factors such as obesity, hypertension, dyslipidemia, and glucose abnormalities^1,2^. Previous longitudinal studies reported that body weight gain was associated with NAFLD among non-obese adults^17–19^, and the risk for NAFLD was significantly and negatively associated with baseline HDL-C levels in non-obese Chinese^41^. Our longitudinal study supports these studies as we found that body weight gain and HDL-C decrease were risk factors for new-onset MASLD, even among healthy individuals at normal weight. Previous studies reported that regular aerobic exercise reduces hepatic fat in non-obese older men, regardless of body weight change^42^, and increases HDL-C level especially in lower BMI individuals^43^. Although not statistically significant between the groups, the MASLD participants had lower physical activity habits (approximately 30%), suggesting that a more active lifestyle is needed to prevent the onset of MASLD in non-obese participants.

The present study identified increased WC as a risk factor for MASLD, which may be attributed to body weight gain. An increase in WC is considered as an indicator of increased visceral fat area, which is an independent risk factor for SLD^44^. A previous cross-sectional study including obese participants reported that WC was an effective discriminator between individuals with and without NAFLD^45^, which is consistent with the results of the present study. As SLD is a condition that is characterized by the accumulation of hepatic fat and the subsequent elevation of endogenous TG levels^46^, it is understandable that elevated TG levels were associated with incident MASLD in this study. Regarding other MASLD criteria, previous cohort studies reported that hypertension was a significant cause of NAFLD in non-obese Chinese adults^47^, and the risk for NAFLD was associated with the incidence of type 2 diabetes in non-obese Koreans^48^. The present study showed that no association between blood pressure and the onset of MASLD, whereas the annual rate of change in FG was associated with a higher risk of incident MASLD only in non-obese women. However, there was no association between MASLD risk and HbA1c, which represents the mean FG level over the previous three months. This suggests that long-term glucose control may not be associated with the onset of non-obese MASLD in the present study.

Cross-sectional studies reported that dietary characteristics are associated with an increased risk of NAFLD in non-obese Korean adults who eat quickly^24^and non-obese Japanese adolescents who consume soft drinks^25^. In our recent cross-sectional study, the prevalences of both NAFLD and MASLD were associated with several dietary characteristics in non-obese Japanese, and the dietary association was similar between non-obese NAFLD and MASLD participants^26^. However, there was insufficient evidence to establish a longitudinal link between non-obese SLD and dietary characteristics. We found that vegetable intake was negatively associated with MASLD onset for non-obese men. For women, a significant association was found in univariate analysis. These findings are consistent with a previous cohort study that reported that vegetable intake was associated with reduced NAFLD risk in both male and female Koreans, which included lean to obese individuals^49^. Furthermore, the study reported that female Koreans showed a decreased risk of NAFLD according to fruit consumption^49^; however, the present study did not identify a significant association between fruit intake and MASLD. These different findings may be due to methodology, as we used a non-quantitative questionnaire. It is well established that dietary fiber consumption has beneficial effects on body weight maintenance, blood pressure, and glycolipid metabolism^50^. A prospective study in the United States found that vegetable fiber intake was a negative predictor of the onset of NAFLD in the general adult population^51^; thus, the adequate consumption of dietary fiber may have prevented the onset of MASLD in non-obese men who often ate vegetables in the present study.

Our study found that the consumption of soy products decreased the risk of non-obese MASLD in Japanese women, and a negative association was also observed in the univariate model for men. A previous meta-analysis demonstrated that soy consumption reduced circulating TG, TC, and LDL-C, whereas it increased HDL-C levels^52^. The favorable effects of soy products on lipid metabolism may be associated with a lower risk of MASLD onset. Another meta-analysis reported that soy consumption reduced serum lipid levels including TG, TC, LDL-C, and HDL-C in postmenopausal women^53^. Because middle-aged and older women were included in the present study, there is a possibility that the sex-specific effects of soy products on lipid metabolism were associated with the lower risk of MASLD in non-obese women. Previous interventional studies reported that soy intake reduced both SBP and DBP in adults^54^. The glycemic effects of soy consumption were not evident in another study^55^, but prospective studies reported that dietary intakes of soy products were negatively associated with the incidence of type 2 diabetes^56^. These beneficial effects of soy products on cardiometabolic risk factors may be a contributing factor to the lower risk of MASLD onset.

In the univariate analyses, some dietary characteristics were associated with MASLD onset in the non-obese participants. Consuming noodles/rice bowls for men and sweet buns/bread with fillings for women were identified as risk factors of MASLD in a previous cross-sectional study^26^; however, these were not found in the present study. As this study excluded middle-aged and older participants with MASLD at baseline, it is possible that these dietary characteristics are associated with an earlier onset of non-obese MASLD. Both often eating an evening meal for men and consuming fried food for women may be associated with excess energy intake, leading to weight gain. Many soft drinks contain high levels of sugars, and prospective cohort studies reported that the consumption of sugar-sweetened beverages increased the risk of obesity^57^. Adjusting for ⊿BMI may be a reason why these significant associations were not found in the logistic analyses.

The present study found that the incidence of SLD was higher in men than in women, which supports the findings of a previous study^40^. The significant association between cardiometabolic criteria and the risk of MASLD was found to be largely consistent across both male and female individuals. The baseline levels of TG were observed to be higher in men than in women, whereas the baseline HDL-C levels were lower in men than in women. This result may be explained by sex difference in lipid metabolism, which is more favorable in women than in men^58^. Favorable lipid metabolism may be associated with a lower prevalence of non-obese MASLD in female individuals. For dietary characteristics, OR and 95% CI for MASLD were similar between vegetable intake for men and soy product intake for women. The differential impact of food on the prevention of MASLD in each sex may be associated with the observed differences in food preference between the non-obese individuals with MASLD and non-MASLD in each sex. While other dietary characteristics were not linked to the onset of non-obese MASLD after adjusting for covariates, female participants demonstrated a higher frequency of consumption of vegetables, fruits, soy products, and sesame/nuts compared with male participants. Additionally, women exhibited lower smoking and drinking habits and higher levels of physical activity than men. This favorable lifestyle may partially explain the lower incidence of non-obese MASLD in women compared with men in the present study.

We followed up participants with onset of NAFLD as a secondary analysis and confirmed that the independent variables were largely similar to MASLD. The strong risk factor of NAFLD onset were BMI gain for both men and women. For dietary characteristics, our multivariable-adjusted model identified a negative association of NAFLD onset with vegetable intake for men and soy product intake for women. These findings suggest that BMI gain and these dietary characteristics are associated with SLD without alcohol abuse in non-obese individuals regardless of cardiometabolic criteria.

This study has several limitations. We used hospital data from annual health checks; thus, there was selection bias. Dietary characteristics were assessed by a non-quantitative method. A recent meta-analysis reported that red meat consumption is a dietary risk factor for NAFLD^59^. However, this study did not evaluate whether the participants frequently consumed red meat. The risk factors for early onset of MASLD could not be evaluated due to middle-aged and older participants not being included. Future research is needed to investigate the quantitative relationship between dietary intake and the onset of non-obese MASLD in the general population. Furthermore, an interventional study is also required to evaluate the quantitative relationship between the predictors of SLD and hepatic fat content using a non-invasive method such as^1^H magnetic resonance spectroscopy.

## Conclusion

BMI gain is a strong risk factor among MASLD criteria in non-obese middle-aged and older individuals. Moreover, the onset of non-obese MASLD is negatively and independently associated with vegetable intake for men and soy product intake for women.

## Electronic supplementary material

Below is the link to the electronic supplementary material.

Supplementary Material 1

Supplementary Material 2

## Data Availability

The datasets used and/or analyzed during the current study are available from the corresponding author on reasonable request.
