# Supplementary figures and images for: BMI gain and dietary characteristics are risk factors of MASLD in non-obese individuals
Source: Sci Rep. 2025 Jan 21;15:2606. doi: 10.1038/s41598-025-86424-x (PMC11751101; doi:10.1038/s41598-025-86424-x)

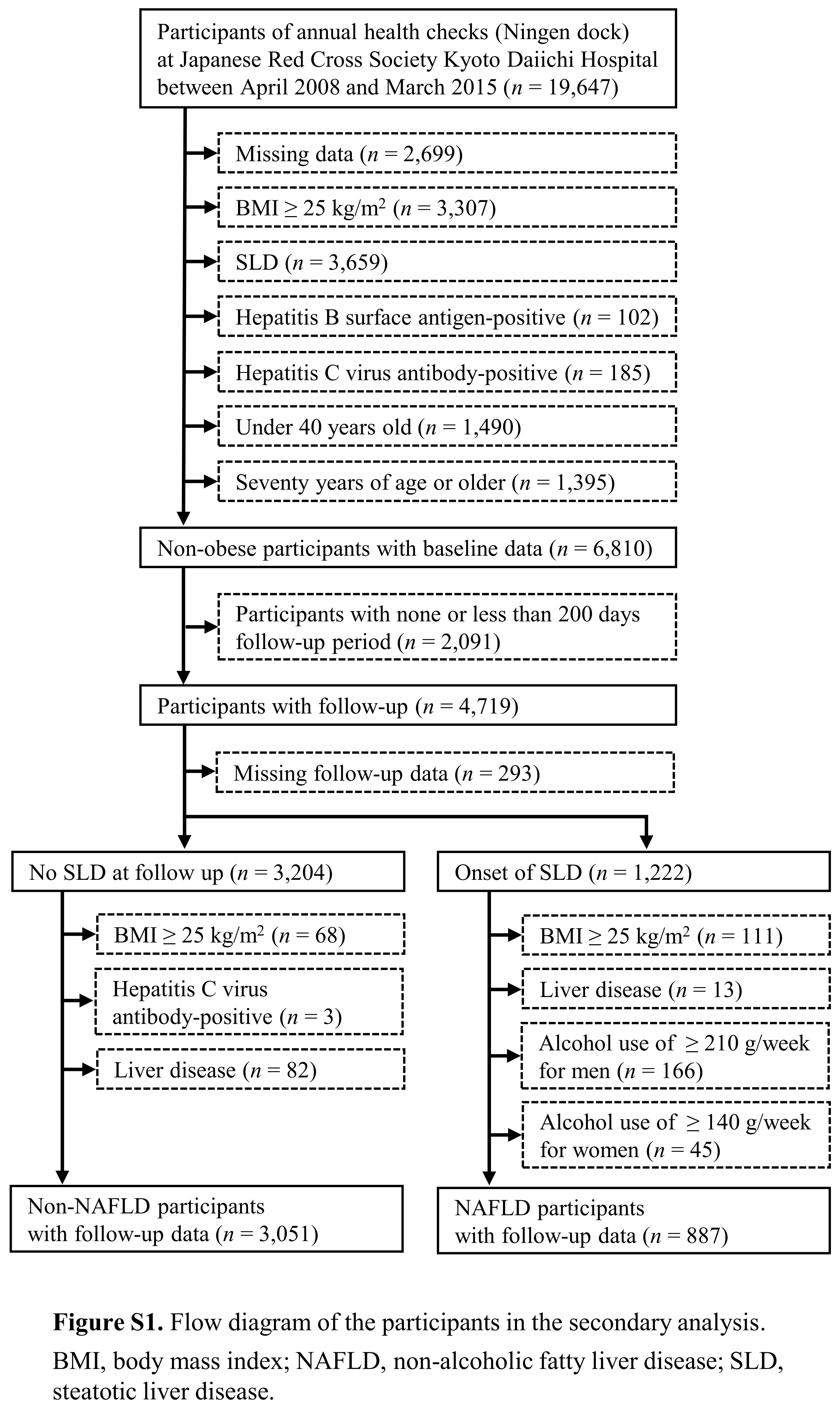

Supplement: Supplementary file 1 — Supplementary Material 1 [file 41598_2025_86424_MOESM1_ESM.tif]
