# Supplementary material for: BMI gain and dietary characteristics are risk factors of MASLD in non-obese individuals
Source: Sci Rep. 2025 Jan 21;15:2606. doi: 10.1038/s41598-025-86424-x (PMC11751101; doi:10.1038/s41598-025-86424-x)
Supplement: Supplementary file 2 — Supplementary Material 2 [file 41598_2025_86424_MOESM2_ESM.pdf]

Table S1. RC per year in MASLD criteria between non-obese individuals with non-MASLD and MASLD.

	Non-obese men (BMI < 25.0 kg/m^2)						p
	Non-MASLD (n = 1,226)			MASLD onset (n = 410)			
RC in BMI, % per year	-0.13	±	1.48	0.73	±	2.13	**<0.001**
RC in WC, % per year	-0.06	±	1.73	0.79	±	2.49	**<0.001**
RC in SBP, % per year	0.62	±	4.64	0.80	±	6.81	0.145
RC in DBP, % per year	-0.05	±	4.88	-0.03	±	6.49	0.176
RC in TG, % per year	2.02	±	17.92	9.00	±	28.56	**<0.001**
RC in HDL-C, % per year	1.51	±	5.98	0.04	±	8.75	**<0.001**
RC in FG, % per year	0.35	±	3.13	0.62	±	4.55	0.077
RC in HbA1c, % per year	0.12	±	3.58	0.33	±	6.56	**0.048**

	Non-obese women (BMI < 25.0 kg/m^2)						p
	Non-MASLD (n =1,980)			MASLD onset (n = 484)			
RC in BMI, % per year	-0.07	±	1.46	0.81	±	1.96	**<0.001**
RC in WC, % per year	-0.10	±	2.29	0.90	±	2.95	**<0.001**
RC in SBP, % per year	0.63	±	4.35	0.46	±	6.56	0.118
RC in DBP, % per year	-0.36	±	5.00	-0.38	±	6.38	0.266
RC in TG, % per year	2.18	±	15.09	6.27	±	20.36	**<0.001**
RC in HDL-C, % per year	1.46	±	4.85	0.22	±	6.53	**<0.001**
RC in FG, % per year	0.52	±	2.63	0.94	±	3.52	**0.038**
RC in HbA1c, % per year	0.24	±	4.28	0.42	±	2.72	**<0.001**

BMI, body mass index; DBP, diastolic blood pressure; FG, fasting glucose; HbA1c, hemoglobin A1c; HDL-C, high-density lipoprotein cholesterol; MASLD, metabolic dysfunction-associated steatotic liver disease; RC, rate of change; SBP, systolic blood pressure; TG, triglycerides; WC, waist circumference.

Data are the mean ± standard deviations. Mann-Whitney U test was calculated between non-MASLD and MASLD participants according to sex. Boldface indicates significance (p < 0.05).

Table S2. Association between RC per year in variables and NAFLD onset in non-obese Japanese men and women.

	Univariate			Multivariable adjusted*		
	OR	(95% CI)	*p*	OR	(95% CI)	*p*
Non-obese men (BMI < 25.0 kg/m^2); NAFLD onset was 384 (25.1%) out of 1,530 men.						
RC in BMI z-score (per year)	1.81	(1.57-2.07)	**<0.001**	1.87	(1.62-2.16)	**<0.001**
RC in WC z-score (per year)	1.68	(1.48-1.92)	**<0.001**	1.72	(1.50-1.97)	**<0.001**
RC in SBP z-score (per year)	0.94	(0.84-1.06)	0.306	0.98	(0.87-1.10)	0.712
RC in DBP z-score (per year)	0.93	(0.83-1.05)	0.229	0.96	(0.85-1.07)	0.443
RC in TG z-score (per year)	1.23	(1.10-1.37)	**<0.001**	1.26	(1.13-1.41)	**<0.001**
RC in TC z-score (per year)	1.01	(0.90-1.13)	0.928	1.01	(0.90-1.13)	0.895
RC in LDL-C z-score (per year)	1.03	(0.92-1.15)	0.634	1.04	(0.92-1.17)	0.537
RC in HDL-C z-score (per year)	0.80	(0.70-0.91)	**<0.001**	0.78	(0.68-0.89)	**<0.001**
RC in AST z-score (per year)	1.12	(1.01-1.25)	**0.044**	1.11	(0.99-1.24)	0.073
RC in ALT z-score (per year)	1.30	(1.16-1.45)	**<0.001**	1.30	(1.16-1.46)	**<0.001**
RC in γ-GTP z-score (per year)	1.18	(1.06-1.32)	**0.003**	1.20	(1.07-1.35)	**0.002**
RC in FG z-score (per year)	1.04	(0.93-1.17)	0.511	1.03	(0.91-1.17)	0.621
RC in HbA1c z-score (per year)	1.02	(0.91-1.13)	0.780	1.03	(0.92-1.15)	0.626
Non-obese women (BMI < 25.0 kg/m^2); NAFLD onset was 503 (20.9%) out of 2,408 women.						
RC in BMI z-score (per year)	1.79	(1.60-2.02)	**<0.001**	1.84	(1.63-2.07)	**<0.001**
RC in WC z-score (per year)	1.48	(1.33-1.66)	**<0.001**	1.46	(1.31-1.63)	**<0.001**
RC in SBP z-score (per year)	0.93	(0.84-1.03)	0.145	0.95	(0.86-1.05)	0.327
RC in DBP z-score (per year)	0.96	(0.87-1.06)	0.457	0.97	(0.88-1.07)	0.521
RC in TG z-score (per year)	1.23	(1.13-1.35)	**<0.001**	1.21	(1.11-1.33)	**<0.001**
RC in TC z-score (per year)	1.02	(0.93-1.13)	0.651	1.01	(0.91-1.11)	0.926
RC in LDL-C z-score (per year)	1.13	(1.03-1.25)	**0.012**	1.11	(1.01-1.23)	**0.032**
RC in HDL-C z-score (per year)	0.78	(0.71-0.86)	**<0.001**	0.79	(0.71-0.87)	**<0.001**
RC in AST z-score (per year)	1.08	(0.99-1.18)	0.102	1.08	(0.98-1.18)	0.108
RC in ALT z-score (per year)	1.17	(1.07-1.29)	**0.001**	1.16	(1.06-1.28)	**0.002**
RC in γ-GTP z-score (per year)	1.14	(1.04-1.25)	**0.006**	1.14	(1.03-1.26)	**0.010**
RC in FG z-score (per year)	1.11	(1.01-1.23)	**0.029**	1.10	(0.99-1.21)	0.069
RC in HbA1c z-score (per year)	1.01	(0.92-1.11)	0.834	1.00	(0.91-1.10)	0.995

*Adjusted for baseline age, BMI, smoking habit, physical activity habit, drinking habit, and medical treatment.

ALT, alanine aminotransferase; AST, aspartate aminotransferase; BMI, body mass index; CI, confidence interval; DBP, diastolic blood pressure; FG, fasting glucose; γ-GTP, γ-glutamyl transpeptidase; HbA1c, hemoglobin A1c; HDL-C, high-density lipoprotein cholesterol; LDL-C, low-density lipoprotein cholesterol; NAFLD, non-alcoholic fatty liver disease; OR, odds ratio; RC, rate of change; SBP, systolic blood pressure; TC, total cholesterol; TG, triglycerides; WC, waist circumference.

The results of non-adjusted and covariate-adjusted logistic regression are shown.

Boldface indicates significance ($p < 0.05$).

Table S3. Association between baseline dietary characteristics and NAFLD onset in non-obese Japanese men and women.

	Non-obese men (BMI < 25.0 kg/m^2)						Non-obese women (BMI < 25.0 kg/m^2)					
	Univariate			Multivariable adjusted*			Univariate			Multivariable adjusted*		
	OR	(95% CI)	*p*	OR	(95% CI)	*p*	OR	(95% CI)	*p*	OR	(95% CI)	*p*
Food preferences												
Vegetables	0.62	(0.49-0.78)	**<0.001**	0.64	(0.50-0.82)	**<0.001**	0.78	(0.63-0.96)	**0.020**	0.86	(0.69-1.08)	0.195
Fruits	0.82	(0.64-1.06)	0.122	0.86	(0.66-1.13)	0.286	0.83	(0.68-1.01)	0.067	0.91	(0.74-1.13)	0.410
Soy products	0.74	(0.59-0.95)	**0.016**	0.82	(0.64-1.06)	0.134	0.65	(0.53-0.79)	**<0.001**	0.69	(0.56-0.86)	**<0.001**
Sesame/nuts	1.11	(0.80-1.54)	0.543	1.16	(0.82-1.64)	0.409	0.74	(0.58-0.95)	**0.018**	0.80	(0.62-1.04)	0.093
Sweet buns/bread with fillings	0.97	(0.73-1.29)	0.820	0.88	(0.65-1.19)	0.413	0.99	(0.78-1.26)	0.948	0.94	(0.74-1.21)	0.646
Sweets	1.26	(0.95-1.67)	0.106	1.33	(0.99-1.79)	0.061	1.03	(0.85-1.26)	0.775	1.01	(0.82-1.24)	0.960
Soft drinks	1.79	(1.29-2.50)	**<0.001**	1.34	(0.94-1.91)	0.107	0.97	(0.66-1.42)	0.883	0.77	(0.52-1.14)	0.190
Food styles												
Noodles/rice bowls	1.29	(1.01-1.65)	**0.044**	1.22	(0.94-1.59)	0.132	0.87	(0.66-1.15)	0.338	0.92	(0.69-1.22)	0.543
Stir-/deep-fried food	1.07	(0.83-1.38)	0.617	1.12	(0.86-1.47)	0.403	1.32	(1.04-1.67)	**0.022**	1.23	(0.96-1.57)	0.101
Simmered/teriyaki food	0.91	(0.71-1.17)	0.464	1.00	(0.76-1.32)	0.980	0.80	(0.66-0.98)	**0.031**	0.88	(0.71-1.09)	0.228
Eating out/ready-made food	1.02	(0.77-1.35)	0.893	0.96	(0.71-1.29)	0.777	1.30	(0.99-1.68)	**0.049**	1.15	(0.87-1.50)	0.329
Dietary behaviors												
Fast eating	1.13	(0.90-1.43)	0.304	1.12	(0.88-1.43)	0.362	1.19	(0.98-1.45)	0.088	1.16	(0.94-1.42)	0.160
Evening meal	1.35	(1.05-1.73)	**0.021**	1.19	(0.91-1.56)	0.198	1.04	(0.85-1.28)	0.702	0.96	(0.78-1.19)	0.725
Consume ≥ 30 foods per day	0.86	(0.58-1.26)	0.428	0.95	(0.64-1.43)	0.821	0.76	(0.59-0.99)	**0.042**	0.88	(0.67-1.15)	0.342

*Adjusted for ⊿BMI, baseline age, smoking habit, physical activity habit, drinking habit, and medical treatment.

BMI, body mass index; CI, confidence interval; NAFLD, non-alcoholic fatty liver disease; OR, odds ratio.

The results of non-adjusted and covariate-adjusted logistic regression are shown. Boldface indicates significance ($p < 0.05$).
